# InGaN-based photoanode with ZnO nanowires for water splitting

**DOI:** 10.1186/s40580-016-0092-8

**Published:** 2016-12-14

**Authors:** Junjie Kang, Vinhquang Dang, Hongjian Li, Sungjin Moon, Panpan Li, Yangdoo Kim, Chaehyun Kim, Hakjong Choi, Zhiqiang Liu, Heon Lee

**Affiliations:** 1 Research and Development Center for Solid State Lighting, Institute of Semiconductors, Chinese Academy of Sciences, Beijing, 100086 China; 2 Department of Materials Science and Engineering, Korea University, Seoul, 137-713 Republic of Korea; 3 Research and Development Center for Solid State Lighting, Institute of Semiconductors, Chinese Academy of Sciences, Beijing, 100086 China

**Keywords:** GaN, ZnO, Nanowire, Photoanode

## Abstract

The water splitting properties of InGaN photoanodes equipped with ZnO nanowires were examined in this study. Over the solar spectrum range, the absorbance exhibited a remarkable increase due to the enhanced light absorption caused by the ZnO nanowires. By varying the ZnO nanowires length, the photo-to-current density of photoanodes was increased from 0.017 to 0.205 mA/cm^2^ at 1.23 V versus reversible hydrogen electrode. Consequently, the incident-photon-to-current efficiency was increased by a factor of 5.5 as the ZnO nanowires growth time increased from 2 to 4 h. The results of this research demonstrate the importance of light absorbance and the surface reaction sites of photoanodes on energy harvesting.

## Introduction

Materials such as Fe_2_O_3_, TiO_2_, ZnO, and InGaN have attracted significant attention in photoelectrochemical applications and solar energy storage because of their suitable band gaps for light absorption [1–4]. Over the past few decades, advances in solar cell and water splitting technologies have focused primarily on structure fabrication and catalyst application [5, 6]. InGaN-based materials have proven to be corrosion-resistant in many aqueous solutions, have been widely investigated, and have been used to produce efficient photoelectric devices with low dislocation density and high internal quantum efficiency (IQE). However, the water splitting mechanism of such devices is not yet fully understood [7–9]. Recently, researchers have attempted to use InGaN-based materials with micro- or nano-structures grown via molecular beam epitaxy (MBE) or metal-organic chemical vapor deposition (MOCVD) for water splitting [10, 11]. By combining GaN materials with other catalysts, such as rare-earth materials, oxides, or metals, the efficiency of GaN materials can be further improved [12]. Surface reaction sites are essential for carrier transportation and GaN materials’ photostability. Pu et al. have shown that Rh/Cr_2_O_3_ nanoparticles equipped InGaN/GaN nanowires with additional charge decay pathways can increase overall water splitting efficiency [13]. However, this efficiency is not comparable to that of materials such as SnO_2_ and TiO_2_, because of the relatively low light absorption ratio of GaN materials [2, 14].

Normally, the light absorption ratio is as low as less than 20% around visible range. Moreover, the GaN material has a relatively long induced carrier lifetime and short carrier diffusion length, which makes the induced current density further lower than other material. The p-doping problem in p-GaN layer also constrains the performance of photoanode, which results in low hole mobility at room temperature. The influence of the light absorbance and surface area of GaN photoanode on photo-to-current efficiency have not been fully investigated as well.

Zinc oxide (ZnO), with a bandgap energy of 3.2 eV, has been reported to be a suitable model semiconductor for solar water oxidation because of its low onset potential and high electron mobility [15]. Numerous strategies have been developed to adapt ZnO to water splitting, including fabrication of multiple semiconductor systems (Si/ZnO core/shell nanowires) to reduce the hole-electron recombination [16] and construction of one-dimensional (1-D) nanostructured ZnO-based electrodes with various morphologies for increased surface area and improved charge transportation and light trapping [17–19]. However, few studies related to GaN and ZnO material combinations for water splitting have been reported.

To increase the efficiency of InGaN-based thin film photoanodes, ZnO nanowires have been adapted to InGaN materials grown by the MOCVD method. The correlation between the efficiency of photoanodes and the ZnO nanowire growth time was studied by measuring the light absorption spectrum, the photocurrent density-versus-voltage (J–V) properties, and the incident-photon-to-current efficiency (IPCE).

## Experiment

### Growth of InGaN-based thin film material

Prior to device fabrication, an Aixtron horizontal MOCVD reactor was used to grow broadband light absorption monolithic InGaN wafers on c-plane (0001)-patterned sapphire substrates. The precursors were trimethylgallium (TMGa), triethylgallium (TEGa), trimethylindium (TMIn), and ammonia (NH_3_). Silane (SiH_4_) and bis-cyclopentadienyl magnesium (Cp2 Mg) were used as n-type and p-type dopants, respectively. Before the deposition of a GaN nucleation layer, the sapphire wafer was thermally cleaned at 1150 °C in a H_2_ atmosphere for 10 min. A 30 nm-thick GaN buffer layer was then grown at 500 °C under a reactor pressure of 650 mbar, followed by deposition of a 3 μm-thick undoped GaN (uGaN) layer (Si-doped 8 × 1018 cm^−3^) at 1030 °C and a reactor pressure of 300 mbar. Individual In_0.2_Ga_0.8_N (3 nm)/GaN (15 nm) quantum well (QW) and quantum barrier (QB) were grown at temperatures of 735 and 815 °C. A thin 0.5 nm In_0.06_Ga_0.94_N wetting layer was inserted before the InGaN well layer, grown at the same temperature as the well. During the QW growth, the V/III ratio was set at 1.15 × 10^4^, the reactor was 600 mbar, and the TMIn flows for the wetting layer and the InGaN quantum dots (QDs) layer were 8 and 68 μmol/min, respectively. The temperature was increased to 920 °C to grow a 20 nm pAlGaN electron blocking layer (EBL) and 200 nm pGaN layer (p-doping 3 × 10^19^ cm^−3^). Additional details are provided elsewhere [20].

### Growth of ZnO nanowire material

ZnO nanowires were produced in an aqueous solution using a two-step process described elsewhere [21]. A spin-coating method was used to prepare ZnO seeds on InGaN-based thin films. Zinc oxide particles were dropped on pre-cleaned InGaN-based thin films. After 30 min, the excess zinc oxide particles were removed using deionized (DI) water. The samples were placed on a hot plate to vaporize the water and increase the adhesion between the zinc oxide particles and the InGaN surface. ZnO nanowires were then grown by placing ZnO-seeded InGaN samples in solutions with 200 ml of water, 1.485 g of zinc nitrate hexahydrate (Zn(NO_3_)_2_ 6H_2_O, 98% purity), and 0.7 g of hexamethylenetetramine (C_6_H_12_N_4_, 99% purity) at 90 °C. To evaluate the growth rate of the ZnO nanowires, different growth times were used for the different samples. The samples were then washed with acetone, ethanol, and DI water for 10 min to remove residual organics and growth solution.

### Characterization of InGaN-based material and ZnO nanowires

Scanning electron microscope (SEM) images of InGaN structures were obtained with a field-emission SEM (S-4300). An acceleration voltage of 1.5 kV and an emission current of 1.5 μA were applied. X-ray diffractometry (XRD) was carried out to investigate the crystal quality of the InGaN samples and ZnO nanowires using the D1 system. The ultraviolet (UV) to visible spectra of the photoanodes were recorded using a Jasco V-650 spectrometer.

### Photoelectrochemical measurements

Curves of the photocurrent density versus voltage (J–V) and the photocurrent density versus time were recorded using an Ivium Technologies system. A 150 W xenon lamp (Peccell), calibrated to 100 mW/cm^2^ with a standard Si solar cell (BS-500BK), was used as the light source. All of the samples were investigated using a three-electrode cell configuration in a 0.5 M/l Na_2_SO_4_ near-neutral electrolyte solution at room temperature. The samples, Pt mesh, and Ag/AgCl electrode acted as working, counter, and reference electrodes, respectively. The potentials versus the Ag/AgCl were transformed into potentials versus RHE. The RHE denotes the reversible hydrogen electrode, which is defined as follows:

$$ {\text{E }}\left( {{\text{vs}}.{\text{ RHE}}} \right) = {\text{E }}\left( {{\text{vs}}.{\text{ Ag}}/{\text{AgCl}}} \right) + {\text{EAg}}/{\text{AgCl }}\left( {\text{reference}} \right) + 0.0 5 9 1 {\text{ pH}}, $$

where E_Ag/AgCl_ (reference) was 0.1976 V and the pH value was 7. The IPCE was conducted at an electrode potential of 1.23 V versus RHE, according to the equation IPCE = 1240I_sc_/λP_in_, where I_sc_ is the current density, λ is the wavelength of the incident light, and P_in_ is the incident light intensity. A standard solar cell was used as the calibration reference. Additional information is provided elsewhere [4].

## Results and discussion

**Fig. 1:**
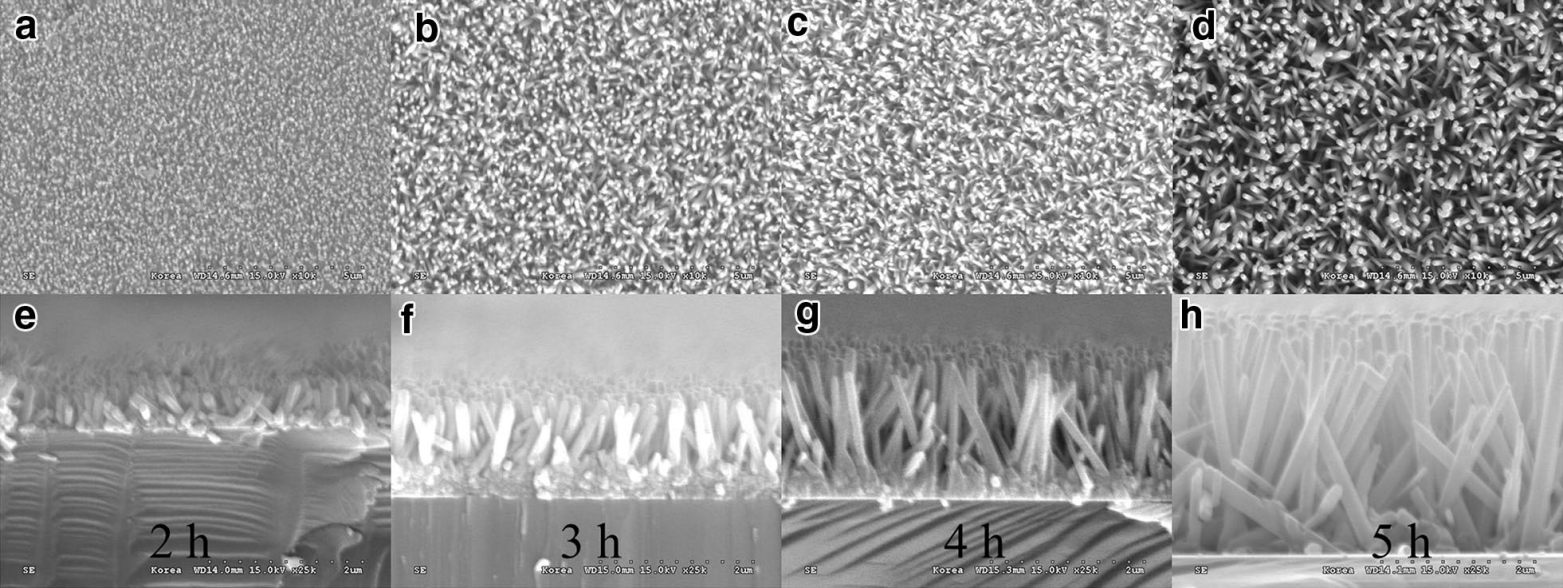
SEM images of ZnO nanowires grown on InGaN-based thin film at growth times of 2, 3, 4, and 5 h. **a**–**d** *Top* views; **e**–**h** *side* views

SEM was used to investigate the morphology of the ZnO nanowires. Figure 1 shows the high-density vertically aligned ZnO nanowires at different growth times. The ZnO nanowire densities were almost the same at all growth times, which suggests that the seed layer distribution determines the ZnO nanowire density, as shown in Fig. 1a–d. The seed layer can be observed easily in the side-view images in Fig. 1e, f. No obvious change was evident for growth times from 2 to 5 h, nor did the diameters of the nanowires change noticeably, whereas the lengths of the ZnO nanowires increased gradually from 1 to 3 μm. After 4 h of growth, the diameter of a single ZnO nanowire was approximately 250 nm. The ZnO nanowire density can be tuned by changing the seed layer density and deposition time (thereby controlling the DI water exposure time).

**Fig. 2:**
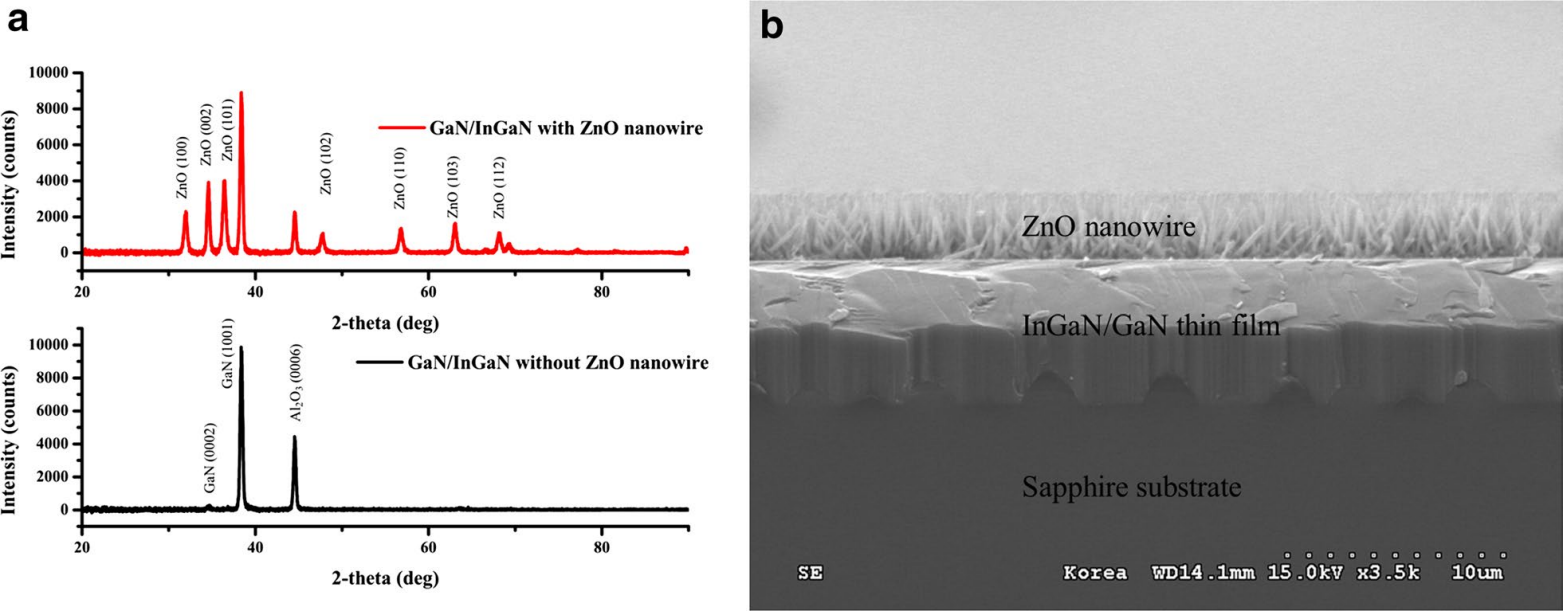
**a** XRD spectra of samples with and without ZnO nanowires on InGaN-based thin film surfaces. **b** Layer information for photoanode with ZnO nanowires

The crystalline properties of ZnO nanowires grown on InGaN-based thin films were investigated by the XRD method. As samples with different growth times had similar XRD spectra, only the XRD spectra with and without ZnO nanowires are presented here (see Fig. 2a). The intrinsic sharp diffraction peaks of ZnO materials can be clearly observed and correspond to the peaks of a wurtzite hexagonal structure. The GaN intrinsic diffraction peaks indicate that the thin film produced was of high quality and was oriented in the (0001) direction. The layer information for this innovative photoanode is shown in Fig. 2b. The layers visible in the figure, from the bottom to the top, are sapphire, InGaN/GaN thin film, and ZnO nanowires.

**Fig. 3:**
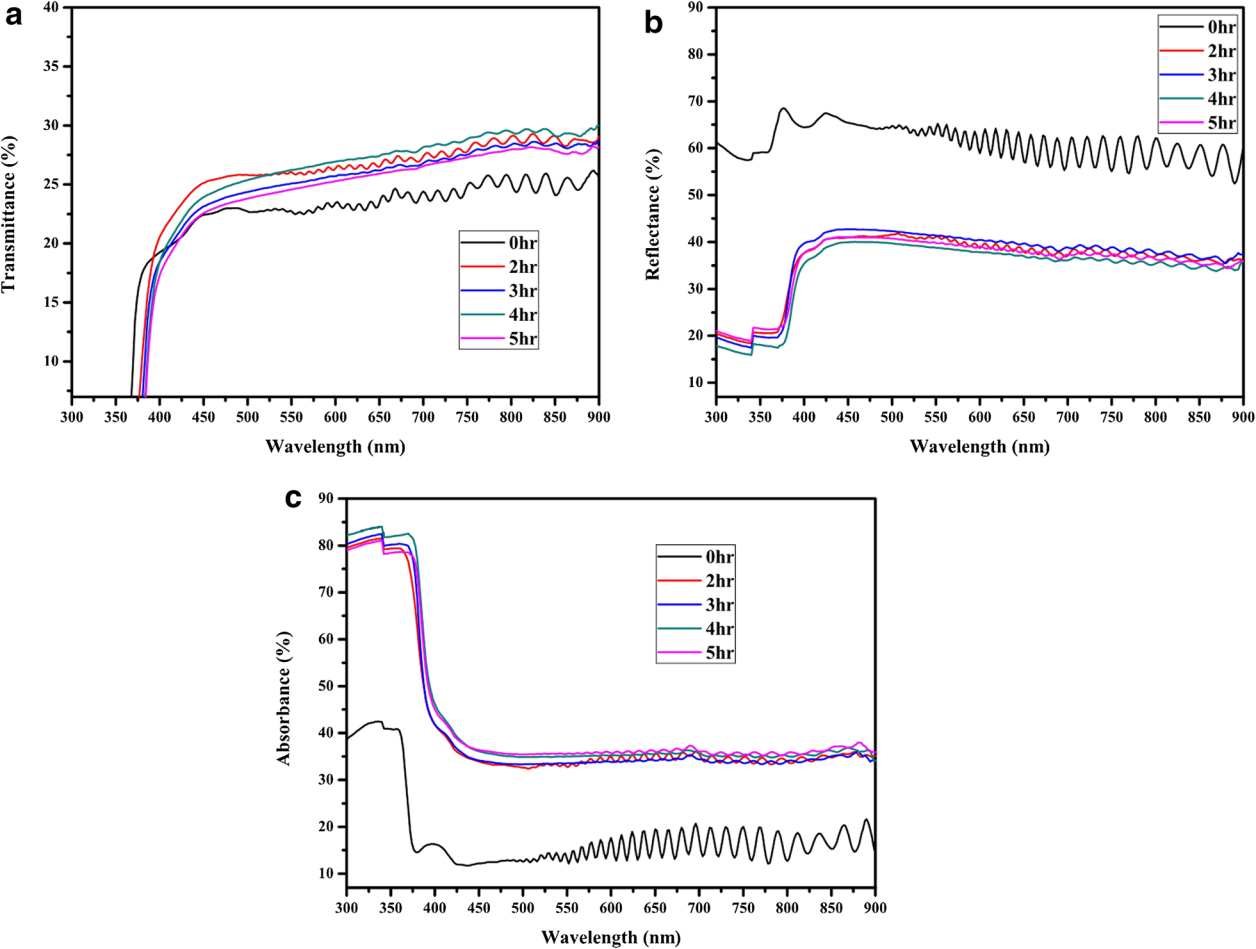
**a** Transmittance, **b** reflectance, **c** absorbance spectra of samples with and without ZnO nanowires, measured as functions of wavelength

The optical spectra of the samples were examined using a Jasco V-650 spectrophotometer. As Fig. 3a shows, the transmittance of the 0 h ZnO growth sample was lower than that of the 2 and 4 h ZnO growth samples over a wavelength of 400 nm, while the transmittance exhibited the opposite trend at wavelengths below 400 nm. The transmittance value increased by approximately 5% on average, and there was an obvious difference between the 2 h ZnO growth and 4 h ZnO growth samples. The reflectance spectra confirmed that, with ZnO nanowires on the surface, less reflective light was observed over the solar spectrum range, as shown in Fig. 3b. As a result, samples with ZnO nanowires exhibited better light absorbance—more than 80% from 300 to 400 nm—than samples without ZnO nanowires, as shown in Fig. 3c. The light trapping effect and UV light absorption induced by ZnO nanowire material together influenced the photoelectrochemical performance of the samples. In addition, ZnO nanowires on InGaN-based thin film surfaces can effectively enhance the reaction sites and carrier diffusion efficiency during the water splitting process. After ZnO nanowire growth, multiple-layer metals of Cr/Pt/Au with thicknesses of 10/100/1000 nm, which are commonly utilized in InGaN-based devices, were deposited to produce ohmic contact on the partly dry etched nGaN surface.

**Fig. 4:**
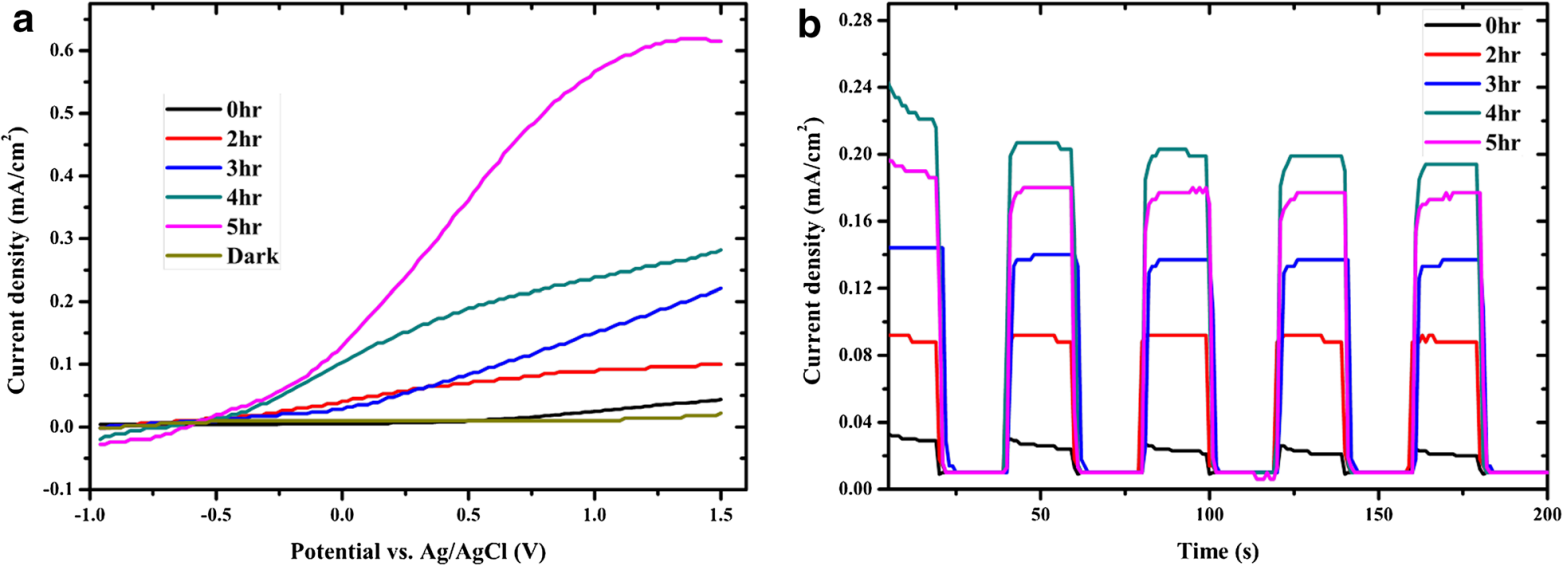
**a** Photocurrent density versus potential for samples with and without ZnO nanowires in 0.5 M/l Na_2_SO_4_ solution under sunlight illumination. **b** Photocurrent density versus time for samples with and without ZnO nanowires under applied potential of 1.23 V versus RHE

The J–V properties of the samples with and without ZnO nanowires were studied under one sun illumination with a sweep rate of 10 mV/s, as shown in Fig. 4a. The dark current density was negligible for all of the samples. The photocurrent density of the bare InGaN-based thin film at a potential of 1.23 V (vs. RHE) was approximately 0.015 mA/cm^2^. For the sample with 2 h of ZnO nanowire growth, the photocurrent density was significantly increased to 0.077 mA/cm^2^ under the same conditions. The photocurrent density was further increased as the ZnO nanowire growth time increased to 4 h. The photocurrent density was enhanced for two reasons. First, ZnO nanowires on InGaN-based thin film surfaces can effectively increase light trapping, which results in greater light absorption of the device. At the same time, ZnO nanowires have an intrinsic light absorption in the UV range. Second, InGaN-based thin films with ZnO nanowires on their surfaces have larger surface areas than bare InGaN-based thin films. More reaction sites are involved in the photo-to-current reaction under sunlight exposure, which also facilitates the energy conversion efficiency.

The photocurrent density versus time (J–T) under an externally applied potential for all samples at 1.23 V versus RHE is shown in Fig. 4b. The photocurrent density of the sample with the 4 h ZnO growth time was approximately 13 times higher than that of the bare InGaN-based thin film photoanode, which is consistent with the previously mentioned J–V results. The enhancement is attributable to the ZnO nanowires’ absorption and light trapping effect, as discussed before. It was observed that as the ZnO nanowire length increased, the photocurrent density of the photoanodes became more stable. As for bare InGaN-based thin film photoanodes, the overshoots caused by separation of photo-generated pairs of electrons and holes were severe. Holes can easily be accumulated at the photoanode surface.

**Fig. 5:**
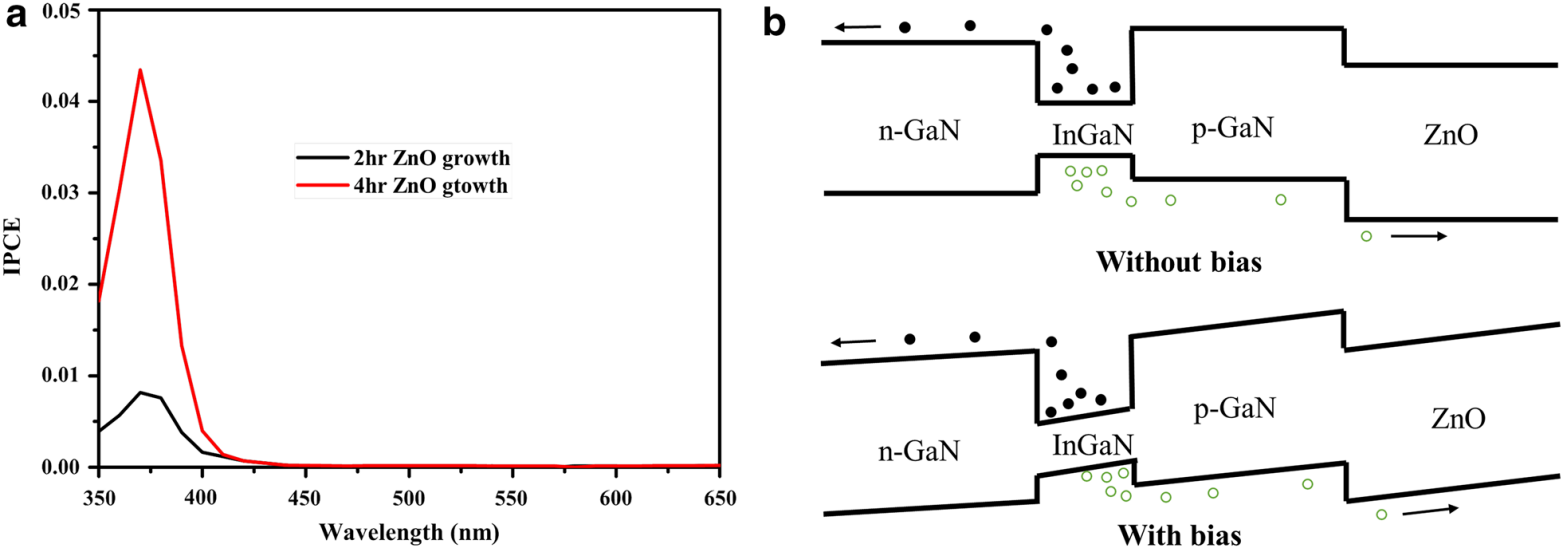
**a** IPCE of samples with ZnO growth times of 2 and 4 h in 0.5 M/l Na_2_SO_4_ solution under applied potential of 1.23 V versus RHE. **b** Simplified band diagram of InGaN-based sample with ZnO nanowires under equilibrium and bias

The IPCE curves of samples with ZnO growth time of 2 and 4 h were measured at 1.23 versus RHE, as shown in Fig. 5a. For InGaN-based thin film with a 2 h ZnO growth time, the IPCE value reached a maximum of 0.8% at a wavelength of 370 nm. For the InGaN-based thin film with a 4 h ZnO growth time, the IPCE value increased to 4.4% at a wavelength of 370 nm. The wavelength absorption range for these two samples was 350–430 nm. Because of the low light absorption rate of the QW region, no distinct IPCE peak was observed at approximately 470 nm, which is consistent with the indium composition of the single QW. The absorbance spectra indicated that the light absorption increased from 60 to 80% around the UV range. However, the photocurrent density and IPCE enhancements were much higher, which means that carrier diffusion and reaction sites also play key roles in the behavior of InGaN-based photoanodes. This finding should be confirmed by other measurement methods in the future. The simplified band diagram is demonstrated in Fig. 5b. Without bias, the generated carriers face large potential when flow from the active region to the P or N electrode separately. Under the bias, the band incline efficiently increases the carriers overflow the potential barriers.

## Conclusions

The photoelectrochemical properties of InGaN-based thin film photoanodes with ZnO nanowires were studied in detail. The photocurrent density of InGaN-based thin films was found to be improved tremendously by the application of ZnO nanowires and to remain stable over long time periods. As a result, the IPCE value was also increased. The efficiency enhancement can be attributed to UV absorption by ZnO nanowires, the light trapping effect, and enlarged reaction sites on the thin film surfaces.
